# Evidence of EBV infection in lymphomas diagnosed in Lusaka, Zambia

**DOI:** 10.11604/pamj.2018.29.181.11847

**Published:** 2018-03-28

**Authors:** Doris Kafita, Trevor Kaile, Evans Malyangu, Rabecca Tembo, Ephraim Zulu, Chrispin Chisanga, Annie Kalonda, Mulemba Samutela, Pascal Polepole, Geoffrey Kwenda

**Affiliations:** 1 Department of Biomedical Sciences, School of Medicine, University of Zambia, Lusaka, Zambia; 2 Department of Pathology and Microbiology, School of Medicine, University of Zambia, Lusaka, Zambia; 3 Department of Pathology, Maina Soko Military Hospital, Lusaka, Zambia

**Keywords:** Epstein-Barr virus, Hodgkin´s lymphoma, Non-Hodgkin´s lymphoma, Molecular detection, Zambia

## Abstract

**Introduction:**

Epstein-Barr virus (EBV) is a ubiquitous virus that infects more than 90% of the world's population, and is implicated in lymphoma pathogenesis. However, in Zambia during the diagnosis of these lymphomas, the association of the virus with the lymphomas is not established. Since most patients with lymphomas have poor prognosis, the identification of the virus within the lymphoma lesion will allow for more targeted therapy. The aim of this study was to provide evidence of the presence of the EBV in lymphomas diagnosed at the University Teaching Hospital (UTH) in Lusaka, Zambia.

**Methods:**

One hundred and fifty archival formalin-fixed paraffin embedded suspected lymphoma tissues stored over a 4-year period in the Histopathology Laboratory at the UTH in Lusaka, Zambia, were analysed. Histological methods were used to identify the lymphomas, and the virus was detected using Polymerase Chain Reaction (PCR). Subtyping of the virus was achieved through DNA sequencing of the EBNA-2 region of the viral genome. Chi square or fisher's exact test was used to evaluate the association between EBV status, type of lymphoma and gender.

**Results:**

The majority of the lymphomas identified were non-Hodgkin's lymphoma (NHL) (80%) followed by Hodgkin's lymphoma (HL) (20%). EBV was detected in 51.8% of the cases, 54.5% of which were associated with NHL cases, while 40.9% associated with HL cases. The predominant subtype of the virus in both types of lymphomas was subtype 1. One of the lymphoma cases harboured both subtype 1 and 2 of the virus.

**Conclusion:**

This study showed that EBV is closely associated with lymphomas. Therefore, providing evidence of the presence of the virus in lymphoma tissues will aid in targeted therapy. To our knowledge this is the first time such data has been generated in Zambia.

## Introduction

Epstein-Barr virus (EBV) is an oncogenic lymphotropic virus that belongs to the γ-herpesvirus family [1]. It is a highly successful ubiquitous virus that infects more than 90% of the world's population, and is associated with several diseases whose incidence differs dramatically in different parts of the world [2]. While infection of EBV is ubiquitous, tumorigenesis only occurs in a small fraction of the infected population, suggesting that the tumorigenic transformation of human cells by EBV involves complex virus-host interactions and other additional co-factors. A compromised host immune condition and a chronic inflammatory microenvironment probably play major roles in mediating the pathogenic actions of EBV in human malignancies [3]. EBV is aetiologically linked to multiple malignancies that include nasopharyngeal carcinoma (NPC) in Southern Chinese people, a high incidence of Burkitt's lymphoma (BL) in sub-Saharan Africa and a high incidence of infectious mononucleosis in teenagers and young adults in Western countries [4]. Each of these exceptional geographical or demographic differences in disease incidence may be accounted for by other cofactors but there has long been interest in the possibility that genetic variation in the EBV in different parts of the world might play a role [5]. EBV is associated with Hodgkin's lymphomas (HL) and some diffuse large B-cell lymphomas (DLBCL) [6]. AIDS patients are at greater risk of developing aggressive lymphomas, and in most cases EBV has been associated with the pathogenesis of these tumours [7]. Older adults and children who are EBV-positive have a poor prognosis, possibly reflecting a poor immune status, which in turn means that these patients may tolerate the disease and its treatment less effective [8]. Making use of EBV association with lymphomas for clinical purposes and therapeutic treatment is of interest [9]. Therefore, the aim of this study was to provide evidence for the presence of EBV in lymphomas diagnosed at the University Teaching Hospital in Lusaka, Zambia.

## Methods

**Study design:** This was a laboratory-based retrospective cross-sectional study on archival formalin-fixed paraffin embedded (FFPE) lymphoma tissue stored over a 4 year period from January 2011 to December 2014 in the Histopathology Laboratory of the University Teaching Hospital, in Lusaka. The University Teaching Hospital is a tertiary referral and teaching hospital with a bed capacity of about 2000. It is the biggest reference hospital and the centre for all histopathology diagnostic work in Zambia.

**Specimens:** The blocks of tissues included cases from all age groups ranging from 9 months to 85 years. The blocks were prepared from tissues obtained from different anatomical sites (anal tissue, right parotid tumour, inguinal lymph node, cervical lymph node, from the nose, ulcerated skin, etc.) from 86 male and 64 female patients.

**Histopathology:** Tissue sections were cut at 6μm on a Shandon Finesse 325 microtome (Thermo Scientific-Shandon, California, USA). Diagnosis of lymphomas was examined by Haematoxylin and Eosin staining of the histological sections which were examined by an experienced pathologist and grouped as Hodgkin's lymphoma (HL) or non-Hodgkin's lymphoma (NHL).

### Detection of EBV and its Subtypes in Lymphomas

**Section cutting:** New blades were used to cut each tissue to avoid contamination during PCR amplification of the viral targets. The sections were then transferred into a separate sterile 1.5ml microfuge tube until required for DNA extraction. Gloves were used at all stages during tissue manipulation.

**DNA extraction:** Up to 30mg of tissue sections were placed in a sterile 1.5ml microfuge tube. DNA was then extracted using the EZNA Tissue DNA Extraction Kit (Omega Bio-Tek Inc, Norcross, Georgia, USA) for paraffin-embedded tissue according to the manufacturer´s protocol. The DNA was eluted in 50μl volumes and stored at -20^o^C until required.

**Detection of EBV DNA sequences:** EBV detection and typing was determined by PCR with specific primers for EBNA3C gene. The sequences and positions of these primers are as follows: EBVD-F, 5'-AGAAGGGGAGCGTGT GTTGT-3' (B95-8 coordinate 87651-87670); EBVD-R, 5'-GGCTCGTTTTTGACGTCGGC-3' (B95-8 coordinate 87803-87784), which yield an amplification product of 153bp for EBV-1 and a product of 246bp for EBV-2. PCR was performed in 25μl using 2.5μl of 5pM of the forward and reverse primers, 12.5μl 2X PCR Master Mix (Thermo Scientific, Hanover, MD, USA), with a final MgCl_2_ concentration of 3mM, 2ul genomic DNA. The reaction mixture was initially denatured at 95°C for 5 min followed by 35 cycles including denaturation at 95°C for 45s, annealing at 56°C for 45s, extension at 72°C for 1min, and finally elongation at 72°C for 10min. Electrophoresis of 5μl of the PCR product was performed on a Tris-Borate-EDTA (TBE) agarose gel (wt/vol) (100V) containing 1μl ethidium bromide (10 mg/ml). A 50bp ladder (Thermo Scientific, Hanover, MD, USA) was used as a molecular weight standard and all gels were visualised using a Biotop Biosens SC-645 Gel Documentation System (Biotech Co. Ltd, Shanghai, China). A human β-actin internal control was used to ascertain the quality of DNA extracted. The following primer pair was used: Forward 5'-GCC ATG TAC GTT GCT ATC C-3' and Reverse 5'-CCG CGC TCG GTG AGG-3'). The following conditions were used with primers: initial denaturation of 94°C for 1 minute, 35 cycles of 95°C for 30 seconds, 62°C for 1 minute, 72°C for 1 minute and 1 final extension cycle of 10 minutes at 72°C . The PCR products were analyzed as described above.

**PCR subtyping of EBV targeting the EBNA-2 Gene:** EBV targets identified as EBV-1 and EBV-2 where then subjected to PCR by amplifying a portion of the EBNA-2 gene. Either with EBNA-2 primers specific for EBV type 1 (EBVD1F: 5'-TCTTGATAGGGATCCGCTAGGATA-3', nucleotide positions 48839-48862 and EBVD1R: 5'-ACCGTGGTTCTGGACTATCTGGATC-3', nucleotide positions 49335-49311 or with EBNA-2 primers specific for EBV type 2 (EBVD2F: 5'-ACTGGATATGAATCCCCTGGGCAG-3', nucleotide positions 48766-48789 and EBVD2R: 5'-GAGTCCTGTACTATCAGAACTACAATG-3', nucleotide positions 49231-49205) were used. Amplification conditions (in 50μl assays) were: Samples were processed for 35 cycles on a GeneAmp System 2700 PCR thermocycler (Applied Biosystems, Foster City, CA, USA) with denaturation at 94°C for 30 sec, annealing at 64°C for 1 min, extension at 72°C for 1 min. Amplified sequences were visualized on a 1.5% agarose gel using standard conditions. A 50bp DNA marker was used as a molecular size marker.

**DNA sequencing of EBNA-2 PCR products:** Prior to sequencing, the PCR products were first purified with the QIAquick Gel Extraction Kit (Thermo Scientific, Lithuania) following the manufacturer's instructions. Forward or reverse linear amplification were performed in 10ml. Linear amplification consisted of 25 cycles of denaturation at 96°C for 10s, annealing at 60°C for 30s and elongation at 72°C for 60s using the iCycler Thermocycler (Bio-Rad, Hercules, CA, USA). Fluorescence-labelled DNA was purified using the ethanol precipitation method. Briefly, the entire extension products were then transferred into 80ml of freshly prepared precipitation solution (3ml of 3M sodium acetate [pH 4.6], 62.5ml of non-denatured 95% ethanol and 14.5ml deionised water), incubated for at least 1hr at room temperature and centrifuged at 14000rpm for 20min. After carefully removing the supernatant, 250ml of 70% ethanol was added to the pellet, vortexed and the contents re-centrifuged at 14000 rpm for 8min. The ethanol was then carefully aspirated and the pellet air-dried for 15min at room temperature. The samples were then analysed on an ABI PRISM 3730XL DNA analyser (Applied Biosystems, Foster City, CA, USA).

**Detection of HIV-1 in lymphomas:** Real time PCR was performed using a TaqMan PCR Core Reagent kit (Applied Biosystems). The forward primer deSK145 was 5'-AGTRGGGGGACAYCARGCAGCHATGCARAT-3', (1358-1387), and the reverse primer deSKCC1B was 5'-TACTAGTAGTTCCTGCTATRTCACTTCC-3', (1485-1512). The TaqMan probes KK-MGB and deKK-MGB were oligonucleotides 5'-ATCAATGAGGAAGCTGCAGAATGGGA-3' (1400-1425) and 5'-ATCAATGARGARGCTGCAGAATGGGA-3' (1400-1425), respectively, coupled with a reporter dye [6-carboxy fluorescein (FAM)] at the 5' end and a non-fluorescent quencher and a minor groove binder (MGB), which is a Tm enhancer, at the 3' end. The 50-μl PCR mixture consisted of 10 μl of extracted DNA solution (50ng/μl); 200nM primers (deSK145 and deSKCC1B); 200nM deKK-MGB probe; 200 μM dATP, dCTP, and dGTP; 400 μM dUTP; 4mM MgCl2; 0.5U of uracil DNA glycosylase; 1.25U of AmpliTaq Gold polymerase; and 1×PCR buffer. Following two thermal steps at 50-μC for 2min and at 95°C for 10min, 45 cycles of two-step PCR at 95°C for 15s and at 60°Cfor 1min were carried out. PCR amplification and data analysis were performed using the ABI PRISM^®^7900HT (Applied Biosystems).

**Data analysis:** Chi square or fisher's exact test was used to evaluate the association between EBV status, type of lymphoma and gender. The level of statistical significance was set at p≤0.05. Data were analysed with GraphPad Prism Software Version 6.0 for Windows (GraphPad Software, San Diego, California, USA). DNA sequences were edited with Ridom Trace Edit Software (Ridom GmbH, Münster, Germany) and analysed by BioEdit Software Version 7.0.9.1 (Ibis Biosciences, Faraday Avenue Carlsbad, California, USA). Identification of the sequences was performed by using the BLAST algorithm [10]. EBNA2 gene sequences were used to construct phylogenetic tree based on the Neighbour-Joining method by using MEGA 6 (Centre for Evolutionary Medicine and Informatics, Tempe, AZ, USA). The reference strain sequences were obtained from the public database, GenBank [10].

**Ethics considerations:** This study was laboratory-based with no direct contact with patients. Ethics approval for the study was sought from the University of Zambia Biomedical and Research Ethics Committee (Ethics clearance number: 017-03-14).

## Results

**Figure 1:**
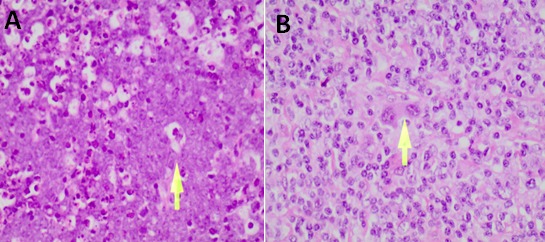
Histopathological features of lymphomas after H and E staining: A) features seen in Burkitt's lymphoma, a NHL, were presence of histiocytes with phagocytosis of nuclear debris, accumulated cellular debris due to increased apoptosis, starry-sky pattern; B) features seen in lymphocyte predominance a HL, included inflammatory cell infiltrate composed of abundant eosinophils, plasma cells, and Reed-Sternberg cell; (Adapted from the University Teaching Hospital- Histopathology Laboratory)

**Figure 2:**
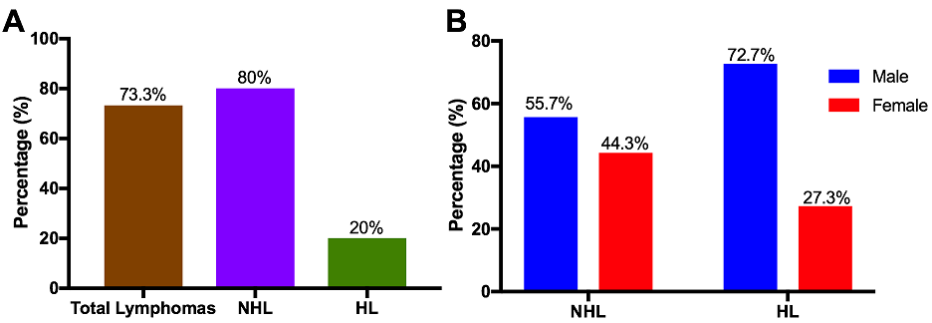
Histological diagnosis of lymphomas and gender distribution: A) total detection of lymphomas and distribution in each type of lymphoma; B) gender distribution in each type of lymphoma

**Figure 3:**
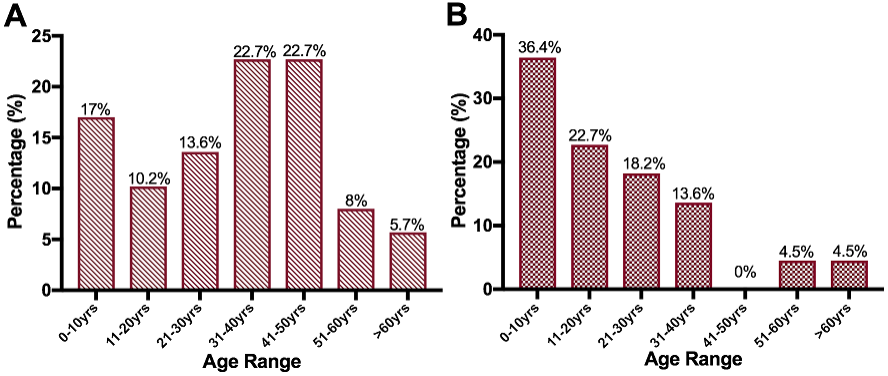
Age distribution in lymphomas: A) NHL cases; B) HL cases

**Histology:** All lymphoma cases were confirmed as either HL or NHL on the basis of different histopathological changes (Figure 1). Of all the tissues examined, 73.3% (110/150) were confirmed as lymphoma cases. Of the 110 lymphomas, 80% (88/110) were identified as NHL, while 20% (22/110) were identified as HL (Figure 2A). NHL affected 55.7% (49/88) males and 44.3% (39/88) females, while HL affected 72.7% (16/22) males and 27.3% (6/22) females (Figure 2 B). In NHL the most affected age group were those aged 31-40 years and 41-50 years age groups (Figure 3 A), while most of the HL cases were mostly in between the ages 0-10 years, followed by the age groups 11-20, 21-30 and 31-40 year age groups (Figure 3 B).

**Figure 4:**
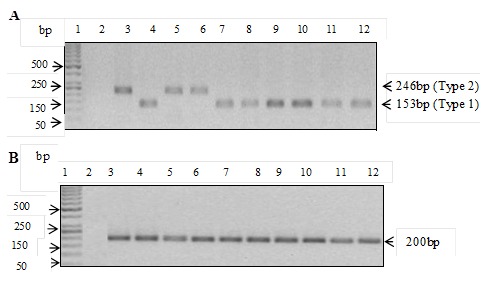
A) EBV detection and subtyping targeting the EBNA 3C region; Lane 1, 50bp DNA marker; lane 2, negative control, lane 3 and 4, positive controls for type 2 and type 1, respectively; lanes 5-12 EBV-positive representative samples; B) actin internal control on selected samples. Lane 1, 50bp DNA marker; lane 2, Negative control; lane 3 and 4, positive controls for type 2 and type 1, respectively; lanes 5-12 representative samples

**Figure 5:**
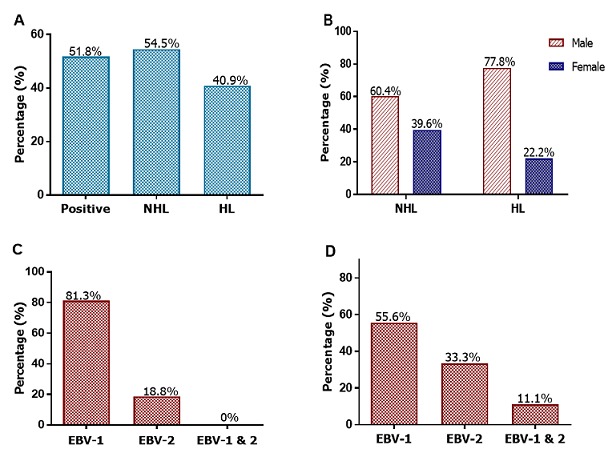
Distribution of EBV positive cases and subtypes: A) EBV DNA detection in all lymphomas and in each subtype of lymphoma; B) distribution of lymphoma cases by gender and EBV-positivity results; C) distribution of EBV subtypes in NHL cases; D) distribution of EBV subtypes in HL cases

**Figure 6:**
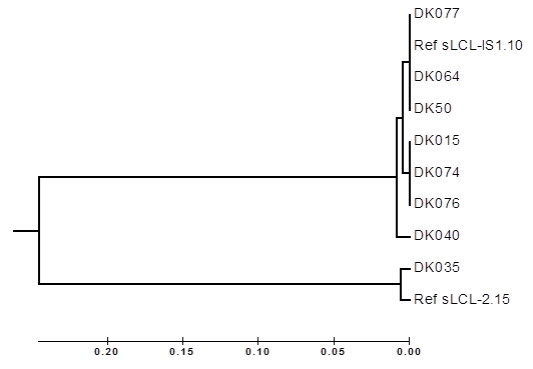
Phylogenetic tree of EBV based on the Neighbour-Joining method. Ref sLCL-IS1.10 is the reference sequence for EBV type 1, while Ref SLCL-2.15 is the reference sequence for EBV type 2

**Detection of EBV DNA and subtypes:** Both EBV subtype 1 and EBV subtype 2 were detectable (Figure 4 A). The quality of DNA used was assessed by PCR amplification of a 200bp fragment of the human β-actin gene which acted as an internal control for the DNA extraction process (Figure 4 B). Of the lymphomas analysed, EBV DNA was detected in 51.8% (57/110) of the cases (Figure 5 A). Of the 88 NHL cases, 54.5% (48/88) were positive for EBV. Of the 20% (22/110) HL cases, 40.9% (9/22) were EBV positive. Statistical analysis of NHL versus HL, p=0.252 (Figure 5 A). In the 48 EBV-positive NHL cases, there were 60.4% (29/48) male patients and 39.6% (19/48) female patients. The difference in the EBV positivity rate between male and female NHL was not statistically significant (p=0.327) (Figure 5 B). In the 9 EBV-positive HL cases, there were 77.8% (7/9) of male patients and 22.2% (2/9) female patients. The difference in the EBV positivity rate between male and female HL was also not significant (p=1.00) (Figure 5 B). In the positive NHL cases, 81.3% (39/48) were of EBV subtype 1, while 18.8% (9/48) were of EBV subtype 2 (Figure 5 C). For the HL EBV-positive cases, 55.6% (5/9) were of the EBV subtype 1, while 33.3% (3/9) were of the EBV subtype 2 (Figure 5 D). Mixed infection was noted in one case of HL. Phylogenetic analysis revealed that the EBV subtypes were segregated into four groups. The first three groups were for EBV subtype 1, whilst the fourth was for EBV subtype 2 (Figure 6). The strains within groups 1 and 2 were indistinguishable, but showed genetic diversity when the two groups were compared. Group 3 only had one strain, and this strain, when compared to the other two groups, revealed that it was distantly related to the other strains in groups 1 and 2. There was only one sequence for the subtype 2 strain available for the comparison as the other sequences were not obtained due to assay failure. For this subtype minor base differences were mainly observed at positions 36890 (A→T), 36891 (C→T), 36893 (A→C) and 37000 (C→T) and 37002(C→G) with regard to the subtype 2 reference strain, Ref sLL-2.15.

**Detection of HIV-1 in lymphomas:** HIV data for the patients was difficult to obtain due to lack of access to patient's records, we only managed for 15 cases of which 4 cases were HIV positive (1 case was EBV type 1 and 3 cases for EBV type 2). Therefore, we decided to find out if any of the remaining lymphoma cases had HIV. After extracting DNA from the formalin fixed paraffin embedded tissue blocks we used Real time-PCR to assess for presence of HIV. None of the 95 lymphoma biopsies indicated HIV integration.

## Discussion

The detection of EBV strains in tissue has been useful in studies involving its transmission and persistence [11]. EBV has been closely linked to the pathogenesis of the majority of lymphomas arising in patients with iatrogenic or congenital immunosuppression [12]. Previous studies have shown that detection of EBV in lymphomas may aid in making correct diagnosis and treatment for EBV-associated lymphomas [13]. Data generated from this study indicate that HL was the most commonly diagnosed lymphoma. This was consistent with studies conducted in Nigeria, Japan, Iraq, and India which reported cases ranging from 60% to 84% of NHL [14-17]. NHL is not a single disease, but a complex and diverse group which encompasses more than 65 different subtypes, and this may explain its predominance when compared to HL [6]. Other reasons may be attributed to the prevailing HIV pandemic and an aging world population due to low immunity [18]. The difference in the distribution of lymphomas in different countries shows that both genetic and environmental factors may play a role in the development of lymphomas [19].

Worldwide, the occurrence of NHL and HL has been reported to be higher in males than in females [20] and this mirrored findings in this study. This male preponderance has also been reported in other countries such as Pakistan, Uganda, Nigeria and India [21-24]. Gender-based differences in immune response are known to contribute to differences in lymphoma development. Males lack factors that have a protective role against lymphoma development in comparison to females. The reduced rates among females can be attributed to the direct effects of oestrogen on lymphoma cell proliferation or by its effect on anti-tumour immune response. In addition, pregnancy and the use of oral contraceptives may also contribute to this protective role in females [25]. It was observed that a high incidence of NHL in both sexes was mostly in the 31 to 40 and 41 to 50 year age groups, the latter being similar to findings of an Indian study in which the most affected age group was from 40 to 50 years [26]. However, this was in contrast to findings in a German study which reported NHL to mainly affect individuals who were aged 55 years and above [27]. In our study, NHL seemed to have afflicted a much younger age group as compared to the German study. This may be attributed to the poor socioeconomic conditions and the predominant immunodeficiency states such as HIV infection prevailing in developing countries such as Zambia [28]. However, it was not possible to link these NHL or HL cases to HIV infection as clinical data for the patients was difficult to obtain due to lack of access to patient's records and none of the lymphoma biopsies showed HIV integration by PCR.

It was, however, observed that the age group mostly afflicted by HL was below 30, with highest peak in those aged between 0-20 years. This finding was consistent with studies carried out in Iraqi and Egypt [15, 16]. In the developed world the disease seems to peak in individuals over 60 years of age [29]. This finding seems to suggest that HL occurs at an earlier age in developing countries than in developed countries [30]. This may be attributed to environmental, genetic and racial factors [23]. Increased risk of HL in the young adult population has also been linked to poor childhood socioeconomic status, which is a major problem among the adolescent population in developing countries [31]. The rate of EBV detection in this study was found to be high, and was similar to a Nigerian study which reported 54.5% EBV-positivity in all lymphomas [32]. This can be attributed to a similarity in the types of lymphomas and subtypes evaluated, as well as the extent of immune suppression of the study population [33]. The EBV positivity rate in NHL was slightly higher than that in HL, although the difference was not statistically significant. This corroborated findings in a Chinese study in which the rate of EBV detection in NHL was also higher (42.6%) than that of HL (26.3%), (p=0.213) [34]. Despite the fact that many studies confirm a strong association between EBV and lymphomas, the detection of EBV has not produced consistent results due to biological heterogeneity of the virus and diversity of lymphoma subtypes [6, 9].

The detection rate of EBV in HL tissues was observed to be low. This finding was consistent with North American and European findings where EBV expression in HL ranged from 20% to 50% [35, 36]. However, our findings were different from observations in other developing countries such as India, Iran, Nigeria, Egypt and Iraq which reported rates ranging from 60 to 90% [15, 30, 37-39]. This difference may be attributed to the fact that the association between EBV infection and HL depends on the geographical location, age, subtype of HL, and the extent of immunosuppression of the study population [39]. A single case of EBV infection (i.e. subtype 1 and 2) was detected, and this was similar to findings in a Spanish study which reported two cases of HL to have mixed infection [40]. Dual infection could reflect immune dysfunction with HIV being a cofactor in the process [40]. However, it was not possible to link this finding to HIV infection as clinical data for the patients was difficult to obtain due to lack of access to patient's records. The rate of EBV positivity for NHL cases in this study was similar to those reported in Brazil, Uganda and Nigeria, which reported rates from 54.5% to 79.8% [9, 22, 32]. This similarity can be ascribed to the anatomical site or types of lymphomas evaluated [33]. The rate of EBV in lymphomas is also dependent on the overall rate of EBV infection in a specific geographical region. For instance, in an Egyptian study, 90% of the EBV-positive cases in lymphomas of head and neck were ascribed to the high frequency of EBV infection in the general Egyptian population [41]. However, the findings in this study are in contrast to those reported in an Iranian study which reported a low rate of EBV in NHL (10%) [33]. This can be attributed to the low prevalence of EBV infection in this population.

There were no significant differences in terms of gender and EBV association. This corroborated studies conducted in China and Uganda [22, 34]. This is not surprising since it is known that EBV is acquired mainly by intimate contact and more than 90% of the world´s population carry EBV as a lifelong asymptomatic infection of B lymphocytes [42]. The predominant subtype of EBV detected in both NHL and HL was subtype 1. This finding was consistent with findings reported in Brazilian and Chinese studies [9, 43]. However, studies conducted in Turkey and Mexico showed that EBV-2 was the predominant subtype [44, 45]. EBV subtype-2 infection has been described to be more prevalent in immunocompromised patients in Western populations, particularly in immunoblastic lymphomas arising in HIV-positive homosexual patients [46]. This difference in the prevalence of the two EBV subtypes can be attributed to the differences in their oncogenicity and the extent of immune suppression of the study population [47].

Phylogenetic analysis revealed that there were four groups of the EBV strains. The first three groups belonged to the EBV subtype 1 category, whilst the fourth one was for EBV subtype 2. The strains within each of group 1 and 2 were indistinguishable from each other. However, there were some major genetic differences between the strains in the two groups. The single strain in group 3, when compared with the other subtype 1 strains, showed it was not closely related to those in the other groups. These findings corroborate those in a Kenyan study [48]. This suggests that inter and intra-genetic variations in EBV genomes exist, and that within a geographical region different EBV genetic strains can coexist [48, 49]. However, a study conducted in the United Kingdom, which examined EBV strains from different geographical locations, showed that strains from the same region clustered together as opposed to those from different regions [49]. This is attributed to genetic differences in the strains circulating in different geographical locations [50]. The single strain in group 4 was the only one amplifiable from the subtype 2 category. The other sequences were not obtained due to assay failure, and thus it was only on available for comparison. When compared to the reference strain, three-base pair differences were observed. This genetic similarity was interesting in that the reference, obtained from GenBank, from originally isolated strain from the United Kingdom. This may suggest that the two strains may have common ancestry. A similar study conducted in Japan also detected only a single case of EBV subtype 2 strain with no mutations when compared to the reference strain [51].

## Conclusion

The study demonstrated that EBV was detectable in the majority of the lymphomas analysed, the rate of detection being higher in NHL than in HL. Therefore, providing evidence for the presence of the virus in lymphoma tissues will aid in targeted therapy. The predominant EBV subtype detected was subtype 1. Phylogenetic analysis of EBV strains revealed genetic diversity amongst EBV type 1, and that the EBV type 2 strain detected showed close genetic relatedness to the reference strain. It may, therefore, be of interest to target EBV during lymphoma diagnosis as this may lead to improved patient outcomes during treatment.

### What is known about this topic

- Studies have shown that EBV is associated with several malignancies including lymphomas. The most common type being EBV type 1.

### What this study adds

- This study provides evidence for the presence of EBV in lymphomas diagnosed at the University Teaching Hospital in Lusaka, Zambia.
